# Anesthesia and brain sensory processing: impact on neuronal responses in a female songbird

**DOI:** 10.1038/srep39143

**Published:** 2016-12-14

**Authors:** G. Karino, I. George, L. Loison, C. Heyraud, G. De Groof, M. Hausberger, H. Cousillas

**Affiliations:** 1 Department of Biotechnology and Life Science, Graduate School of Engineering, Tokyo University of Agriculture and Technology, 2-24-16 Naka-cho, Koganei-shi, Tokyo, 184-8588 JAPAN; 2 Department of Pediatrics, Faculty of Medicine, Saitama Medical University, 38 Morohongo, Moroyama-cho, Iruma-gun, Saitama, 350-0495 Japan; 3 Research Fellow of the Japan Society for the Promotion of Science, 5-3-1 Koji-machi, chiyoda-ku, Tokyo, 102-0083 Japan; 4 CNRS- Laboratoire d’Ethologie Animale et Humaine, UMR 6552 Université de Rennes 1-, 263 av. de Général Leclerc 35042 Rennes cedex, France; 5 Université de Rennes1 - Laboratoire d’Ethologie Animale et Humaine, UMR 6552 - CNRS, 263 av. de Général Leclerc 35042 Rennes cedex, France; 6 Bio-Imaging Laboratory, University of Antwerp, Universiteitsplein 1, B-2610 Wilrijk, Belgium

## Abstract

Whether anesthesia impacts brain sensory processing is a highly debated and important issue. There is a general agreement that anesthesia tends to diminish neuronal activity, but its potential impact on neuronal “tuning” is still an open question. Here we show, based on electrophysiological recordings in the primary auditory area of a female songbird, that anesthesia induces neuronal responses towards biologically irrelevant sounds and prevents the seasonal neuronal tuning towards functionally relevant species-specific song elements.

These results demonstrate a clear impact of anesthesia on sensory and cognitive processes. They demonstrate the need to better understand the actions of these drugs. They also open new lines of thought on the differentiation between sensory basic processing and the brain processing of behaviorally relevant items that require high vigilance.

The question of the potential impact of anesthetics on brain activity and cognitive processes is a highly debated issue for both clinical reasons and research purposes^1^. With the assumption that response properties of central neurons are not changed, most studies on sensory processing in animals have been performed under anesthesia^2^. Nevertheless, studies on auditory processing at different brain levels, and with different anesthetics, converge to show altered neuronal responses under anesthesia, as demonstrated in both humans and animals by imaging methods in humans or electrophysiological recordings in animals, especially for higher cognitive functions involving telencephalic structures^1^,^3^,^4^,^5^,^6^. For example, in songbirds, auditory responses in the higher integrative parts of the vocal control system (HVC and RA) are clearly state dependent^7^,^8^,^9^,^10^. However, at lower integrative levels such as the primary auditory area (Field-L), results from different studies are controversial. For Heinke & Koelsch^1^, the neural processes at the level of the primary auditory cortex would remain intact during sedation while cognitive processes would be affected. In animals, sound processing may thus depend upon its “behavioral significance” that may require a different level of vigilance^3^. Gaese & Ostwald^2^ and Zurita *et al*.^11^, using pure tones, found that anesthesia (Equithesin) increased neuronal phasic on responses but also changed the neurons’ frequency tuning. Few studies have been performed on the processing of species-specific sounds, that is “complex sounds characterized by time-varying amplitude and spectral features”^3^,^6^ of high behavioral significance, under both awake and anesthetized states. However, they led to controversial results. Some showed a strong impact of anesthesia on neuronal selectivity in mammals and birds: no response to some stimuli when awake, to others when anesthetized; changes in neuronal preferences and hemispheric specialization of sound processing^3^,^12^,^13^,^14^,^15^. Others, mostly in birds, led to the conclusion that anesthesia did not affect neuronal tuning^4^,^5^,^16^,^17^. Some authors suggested that songbirds may differ from Mammals^5^ but the fact is that studies performed on songbirds and using the same anesthetics reached also the opposite conclusion^6^,^10^. The reasons for these opposite results remain unknown, but several hypothesis may explain these results. For example, procedure differences often associated with low sampling (in terms of both neuronal sites and individuals), or Interspecific differences of the songbirds used in these experiments. European Starling and zebra finch two species often used in these studies have different vocal behavior, there is a strong adult vocal plasticity in Starlings but not in zebra finch. Another aspect is the type of stimulus used. Electrophysiological data have long shown that neurons may be tuned to complex meaningful vocalizations^18^,^19^,^20^ rather than mere acoustic properties. However, whether anesthetics may also affect the tuning properties of central neurons remains under debate, despite of the fact that this is a major issue: a better knowledge of the effects of anesthesia on neuronal properties is essential as otherwise anesthesia could lead us to potential major errors of interpretation. One could hypothesize that responses to functional social classes based on complex acoustic combinations require higher attentional states than mere responses to simple parameters.

In order to test this hypothesis, we compared the responses of Field L (primary auditory area) neurons in anesthetized and awake-restrained female starlings while broadcasting behaviorally relevant species-specific sounds. In an earlier study, we were able to demonstrate that Field L neurons of awake adult female starlings are tuned to species-specific song elements that are produced by both males and females (Class II and Class III songs; Fig. 1), showing a seasonal plasticity with more responses to non-sexual male song elements outside the breeding season, but more towards sexual song elements (high pitched trills) at breeding time^15^. Here, we tested the responses of auditory neurons of anesthetized females, using exactly the same procedure, both at breeding and non-breeding times. These song elements have been shown to play a major role in the social^21^ and sexual^22^ life of female starlings and therefore are expected to potentially require more cognitive type processes and hence more attention than responses to artificial sounds. We hypothesized that, because of the decrease of attention induced by anesthesia, the processing of these behaviorally relevant species-specific sounds could be more affected than that of artificial sounds.

Electrophysiological recordings of 7920 neuronal sites in Field L (both hemispheres) from 16 anesthetized adult female starlings (3856 neuronal sites) were performed, using exactly the same procedure (systematic recording throughout Field L using 4 microelectrodes^20^) as those performed on awake birds (4064 neuronal sites from 10 birds), with which they were then compared (data already published^15^). Eight birds were recorded during the breeding season and 8 outside. Anesthesia was performed using Ketamine-Medetomidine at a subclinical level. Because it does not require respiratory assistance, ketamine is widely used in studies on mammals and birds, in particular for electrophysiological recordings (e.g. ref. 21). The sounds broadcast were artificial sounds (white noise and 0.5, 1, 2, 4 & 8 kHz pure tones) and song elements corresponding to different functional classes^21^,^22^ (Fig. 1). Class-I songs are Sturnus loud whistles produced only by males and are typical of male-male interactions. Class-II songs are whistles produced by both males and females: they characterize an individual but may also characterize social affinities between individuals (vocal sharing); Class-III songs are composed of long continuous successions of motifs (standardized repeatable groups of notes) produced by both males and females: they are individual specific or shared by close social associates, but two motif types are species-specific: the -clicks, a particular sound structure present all year round in the male warbling song and the -high pitched trills that are produced at the end of the warbling sequence, with high intensity and a wing waving display; they are more frequent in the song of unmated males seeking a sexual partner^21^,^22^.

Among the recorded neuronal sites, 28.66% were responsive to the stimuli used, then all analysis were done on these responsive sites. Since there were no statistical differences between hemispheres (Four-way repeated measures ANOVA, interaction with condition, F < 0.001, p = 1; interaction with season, F < 0.001, p = 1; interaction with Class, F = 2.12, p = 0.06), data from both hemispheres were pooled.

The class-III warbling individual motifs elicited more responses (almost 30% of the responsive neuronal sites) than any other stimuli both in the non-breeding and breeding seasons (ANOVA, F = 32.92 p < 0.0001) while the clicks elicited the least neuronal responses (Fig. 2). This was close to the responses exhibited by the awake females in the non-breeding season, although there were less responses towards the class-III motifs (Tukey’s HSD, p = 0.0048) and more responses towards the artificial sounds in the anesthetized (19.43%) than in the awake (5.62%) females (Tukey’s HSD, p < 0.0001). Clear differences occurred however in the breeding season when the anesthetized birds differed from the awake starlings: they showed no increase of neuronal responses towards the trills, hence the sexual song elements. In fact, the anesthetized females did not show any seasonal pattern contrarily to the awake females, which showed significantly higher responses to the trills (Fig. 3, Tukey’s HSD, p < 0.0001).

Indeed, there were strong interactions between stimulus class and condition (F = 10.943, p < 0.0001) and between stimulus class, condition and season (F = 11.335, p < 0.0001).

The analysis of anesthesia effect performed separately for each season revealed significant changes only at the non-breeding season (Fig. 4). There was an atypical increase of responses towards the artificial stimuli (Tukey’s HSD, p < 0.001) but presenting a high interindividual variability and a decrease of responses to socially relevant class III motifs (Tukey’s HSD, p = 0.004).

This is a clear demonstration, with such a large sample of neuronal sites (7920) and individuals (26 birds), that anesthesia may profoundly affect neuronal selectivity in a primary sensory area: neuronal preferences towards behaviorally significant auditory signals were diminished while responses towards non-significant sounds increased. Such differences may change completely the understanding of sensory but also cognitive processing in central areas. Another striking result was the loss of the seasonal plasticity in neuronal preferences. As the seasonal variation in neuronal preferences appeared only in the awake condition. This means that the vigilance state may strongly contribute to the seasonal neuronal preferences in the auditory region.

Our results seem contradictory with other studies made in the Field L and midbrain of zebra finch^5^,^16^ or in the CMM of starlings^17^, which found no differences between awake and anesthetized conditions. Furthermore, recently Caras *et al*.^23^ have shown seasonal differences on sound level processing under urethane anesthesia on white crowned sparrow females (but not in males). These opposite results may be explained by interspecific differences that depend on the socio-ecological conditions of the bird species. European starling exhibit seasonal variations in vocal production and learning while species like zebra finch do not. Then, the anesthetic effect may be seen only on species with seasonal plasticity. The different anesthetics used in these studies could also explain these contradictory results. They used urethane^16^,^17^ or equitesin^5^ that both increase GABA receptor activity^24^ producing a general inhibition while we were using a mixture of ketamine that alters NMDA receptor in all cortex and medetomidine that is an agonist of α2 adrenergic receptors. Medetomidine can explain our results; the activation of α2 adrenergic receptor alters norepinephrine that enhances auditory coding of complex stimuli in songbirds^25^. Ketamine should have the same kind of influences on cortical neurons than urethane. Ketamine decreases cortical activity acting directly on glutamate receptor while urethane does it indirectly increasing GABAergic inhibition.

However, earlier studies have shown differences in neuronal preferences under urethane anesthesia^13^,^14^, suggesting at that time already that neuronal tuning could be affected by anesthesia. These results however reinforce those of Syka *et al*.^3^ and Huetz *et al*.^12^ in Mammals, contributing strongly to the idea that anesthetics may mainly affect sensory elements that show relevance and require potentially more than a basic sensory processing. Vigilance and attention may be required in order to produce the right behavioral responses towards some particular meaningful vocalizations. Maybe anesthesia may affect “attention units” such as those described in cats, which respond to sounds only if they are associated with some attentional focus of the subject^26^. Interestingly, there might be sexual differences in the effects of anesthesia on seasonal plasticity: female white crowned sparrows are affected by anesthesia but not males^23^ while male starlings are affected but not females^27^. Further studies are clearly needed to understand the relationship between anesthesia, brain adult plasticity and sex. One possibility to explore further is that the findings here are specific to female songbirds because seasonal variations in (neuro-)estrogens can contribute to neuronal plasticity^28^ and modulate the number of catecholaminergic fibers^29^ in the auditory regions. Understanding precisely what aspects of sensory and cognitive processing are affected by anesthesia might lead to new discoveries on the fine processes involved also in the awake state.

## Materials and Methods

### Animals

Sixteen wild-caught adult female European starlings were used for this study. These birds had been caught as adults (as shown by their plumage characteristics^30^; during their fall migration along the Normandy coast (north of France), in October 2012 (i.e. 2 years before the beginning of the experiment). From capture until the experiment took place, they were kept together in a mixed sex group (with other males and females caught at the same time) in an outdoor aviary. The absence of nestboxes prevented breeding to occur and males to display sexual behaviors^31^. Two weeks before the experiment, the birds were placed 2 weeks before in an indoor aviary under artificial light following the natural photoperiod.

Eight females were recorded in the non-breeding season (October-November 2014) and 8 other females during the breeding season (April-May 2015), all under anesthesia (see further). The observation of the beak’s color (yellow at breeding time, dark at non-breeding time) ensured that the birds were effectively in the corresponding internal state^32^,^33^,^34^.

The data obtained from these birds were compared to those obtained from birds (N = 10 females) tested earlier while awake-restrained^15^. These birds had been caught earlier in the same site and had been maintained and tested in exactly the same conditions.

The experiments were performed in France (licence #D35-238-15, issued by the departmental direction of veterinary services of Ille-et-Vilaine) in accordance with the European Communities Council Directive of 22 September 2010 (2010/63/UE). The experimental protocols were approved (#2015091414124239) by the local Ethic Committee (Comité Rennais d’Ethique en matière d’Expérimentation Animale (CREEA)).

### Auditory Stimuli

**Figure 1:**
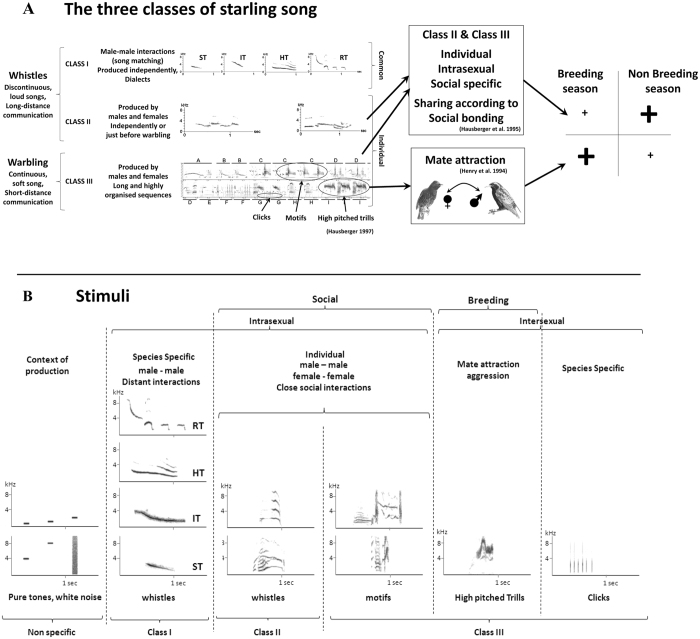
The different classes of starling song (**A**). Class I whistles are species specific, produced only by males and used for distant interactions. They are subdivided into four whistle types: Inflection (IT), Harmonic (HT), Simple (ST) and Rhythmic (RT) themes. Class II whistles and Class III warbling motifs that are produced by both sex are individual and used in a close social context in non-breeding season. The song sharing of Class II and Class III reflects the social organization. At the breeding season the song rate increases with a higher proportion of Class III motifs among which high pitched trills that become predominant are used by unmated males to attract females. (**B**) Sonograms of the auditory stimuli used in the present study. These stimuli are identical to the ones used in the awake study (Cousillas *et al*.^15^). Non-specific sounds and species-specific songs chosen for their behavioral relevance have been used in this work. Pure tones and white noise were used as non-specific stimuli. The 4 themes of Class-I whistle (IT, HT, ST and RT); Class-II individual-specific whistles and class-III individual motifs. Class-I stimuli were one exemplar of each of 4 themes^21^; class-II songs were 2 exemplars of individual whistles; class-III songs were 2 exemplars of individual motifs, one high-pitched trill and one click motif. All these stimuli were produced by males unkwon for the recorded females.

Auditory stimuli were similar to those used in the awake condition^15^: artificial non-specific sounds (white noise and 0.5, 1, 2, 4 & 8 kHz pure tones) and songs (Fig. 1) chosen for their behavioral relevance^35^. Class-I that are species-specific loud whistles produced only by males and that are the bases for male-male interactions and dialectal variations. Class-II whistles that are produced by both males and females characterize an individual but may also characterize social affinities between individuals (song sharing). Class-III warblings that are produced by both males and females are composed of long continuous successions of motifs (standardized repeatable groups of notes). They are individual specific or shared by close social partners but two motifs are species-specific: the -clicks, common in all male songs all year round and the -high-pitched trills that occur at the end of the warbling sequence are produced with high intensity, wing waving display and are more frequent at breeding time especially in unmated males seeking a sexual partner^21^,^22^,^36^.

The stimulus set was made of these artificial non-specific sounds and exemplars of the 3 classes of songs (Fig. 1). Although no neuronal adaptation was reported in the Field L using this kind of stimulus set^18^, the stimuli were broadcast with intervals of at least 300 ms in order to avoid any neuronal adaptation problem between the stimuli. The sequence of stimuli set was determined randomly and then the same sequence was repeated 10 times at each recording site.

### Electrophysiological recordings

Before the neurophysiological experiments, a stainless-steel well was implanted stereotaxically on the bird’s skull under isoflurane anesthesia (0.4 l/min of carbogene −95% O2–5% CO2 - saturated in isoflurane and 0.6 l/min of carbogene^15^). The center of the implant was located precisely with reference to the bifurcation of the sagittal sinus at 2.5 mm rostral and 1 mm in the left hemisphere. This position allowed the introduction of the electrodes in both hemispheres. After implantation, the birds were allowed to rest for 3 days in individual cages. During this period, they could hear but not see each other. They were kept under natural photoperiod throughout the study. During the electrophysiological recordings, the well was used for head fixation and as the electrode reference. Before the first recording session, the bone was removed to allow electrode introduction in both hemispheres.

All recordings were made using the same recording setup at a temperature of about 20 °C and relative humidity of about 30%. Neuronal activity was recorded systematically throughout Field L during the broadcast of every acoustic stimuli, using the same approach as George *et al*.^37^. During recordings, birds were anesthetized with a 4 ml/kg mixture composed of 5 ml Medetomidine (1 mg/ml), 0.25 ml Ketamin (50 mg/ml) and 5 ml saline solution. The final dose was 1.82 mg/Kg of Medetomidine and 4.55 mg/kg of Ketamin. The anesthetic product was injected using an intramuscular way in the thoracic muscles. The recordings lasted about 6 h (+/−10 min.). In order to maintain the anesthesia level, we injected every 2 h a third of the first dose. These injections were done systematically while the bird was still anesthetized. This was the only difference with the earlier study where instead of being anesthetized the birds were awake and kept in a jacket in order to limit their movements.

A head holder was used to maintain the bird’s head in a constant and stable position. We used an array of four microelectrodes (two in each hemisphere) made of tungsten wires insulated by epoxylite (FHC). Electrode impedance was in the range of 5–6 MΩ each. These electrodes spaced 1.2 mm apart in the sagittal plane and 2 mm apart in the coronal plane. Recordings were performed in one sagittal plane in each hemisphere. These planes were precisely located with reference to the bifurcation of the sagittal sinus: 2.5 mm rostral and 1 mm in each hemisphere. These coordinates ensured that recordings were made in Field L centered on the L2 sub-area described by Capsius and Leppelsack^38^ and Cousillas *et al*.^39^. The precise brain areas concerned have been described in detail in a recent paper on starlings’ brain atlas^40^ which helped us guarantee the location of our recordings. Furthermore, the artificial non-specific stimuli composed by pure tones and white noise allowed us to assess the presence of the tonotopic organization that is characteristic of Field L^18^,^39^. This procedure allowed us to avoid a postmortem analysis of the recording sites, as required by the 3 R ethical considerations and to keep the birds alive for future behavioral experiments. For a similar ethical reason the awake and anesthetized conditions were not recorded in the same birds. The awake experiment was already published and in order to respect the “R” of “Reduction” the ethical committee does not allow to perform twice the same experiment using a second group of birds, thus only the anesthesia condition could be performed. Recordings in the left and right hemispheres were made simultaneously, at symmetrical locations. The recording planes were at the same location for all birds. Recordings were performed at 30 to 40 sites along the path of an electrode penetration. Three penetrations could be done during a 6 h session. Penetrations within one recording plane were 200 μm apart. For each penetration, recordings started 600 μm below the brain surface, at a site that gave no auditory response, and continued, every 200 μm, until no response was obtained in both outermost penetrations. The dimensions of the recording plane were 2.4 mm caudo-rostral and 3.6 mm dorso-ventral (8.64 mm^2^ area).

### Data analysis

**Figure 5:**
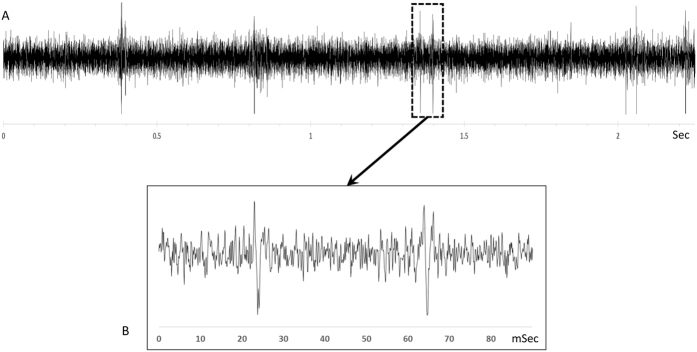
Example of typical raw waveform (**A**) and spike waveform (**B**) of extracellular recordings in Field L. The signal to noise ratio varied from 3 to 6.

Again data analysis was the same as in Cousillas *et al*. (2013)’s study^15^. Spike arrival times were obtained (with a temporal resolution of 0.1 ms) by thresholding the extra-cellular recordings with a custom-made time- and level-window discriminator^37^. The signal to noise ratio varied from 3 to 6 (Fig. 5). Single units or small multiunit clusters of 2–4 neurons were recorded in this manner. Since several studies found that analyses resulting from single and multi-units led to similar results^41^,^42^, the data from both types of units were analyzed together.

**Figure 6:**
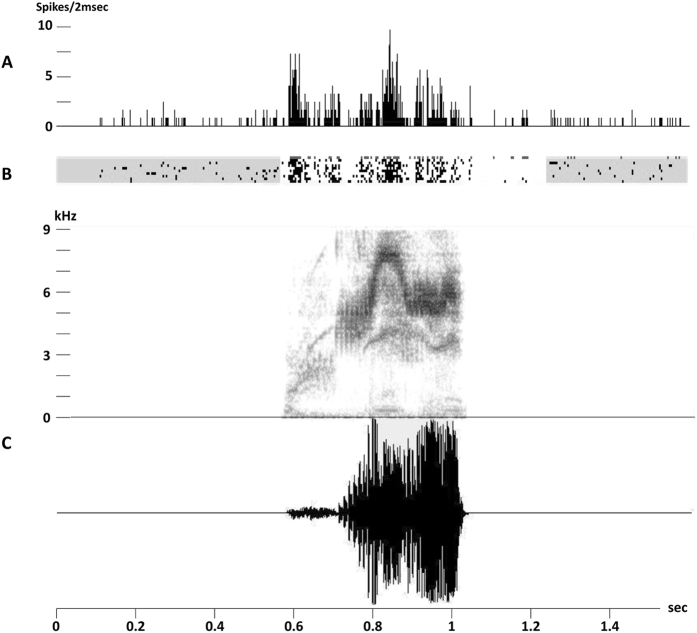
Example of auditory response of field L responsive site to the high-pitched trill. (**A**) Post-stimulus time histogram (PSTH) cumulated over the 10 stimulus sequence repetitions (2 ms time bins); (**B**) raster plot of the neuronal activity related to the 10 stimulus repetitions; (**C**) spectrogram and oscillogram of the high-pitched trill.

The computer that delivered the stimuli also recorded the times of action potentials and displayed on-line rasters of the spike data for the 4 electrodes simultaneously (Fig. 6). At each recording site, spontaneous activity was measured during 1.55 s before the presentation of the first stimulus of each sequence, which resulted in 10 samples of spontaneous activity (that is a total of 15.5 s).

Neuronal responsiveness was assessed as in George *et al*.^16^ by comparing activity level (number of action potentials) during stimulation and spontaneous activity using binomial tests. Only responsive sites were further analyzed by calculating the proportion of sites responding to each stimulus and to each class of stimuli. The mean values calculated for individual birds (n = 10) were then used for statistical comparisons.

### Statistical analyses

Four-way repeated-measures ANOVAs and Tukey HSD tests (R 2.15.2, R Foundation for Statistical Computing) were performed to test for potential differences between the two conditions (awake or anesthetized), the two hemispheres, the two seasons and the different classes of stimuli, as well as the conditions (anesthetized/awake) Data were normalized using an arcsin square-root transform.

## Additional Information

**How to cite this article**: Karino, G. *et al*. Anesthesia and brain sensory processing: impact on neuronal responses in a female songbird. *Sci. Rep.* **6**, 39143; doi: 10.1038/srep39143 (2016).

**Publisher's note:** Springer Nature remains neutral with regard to jurisdictional claims in published maps and institutional affiliations.

## Figures and Tables

**Figure 2:**
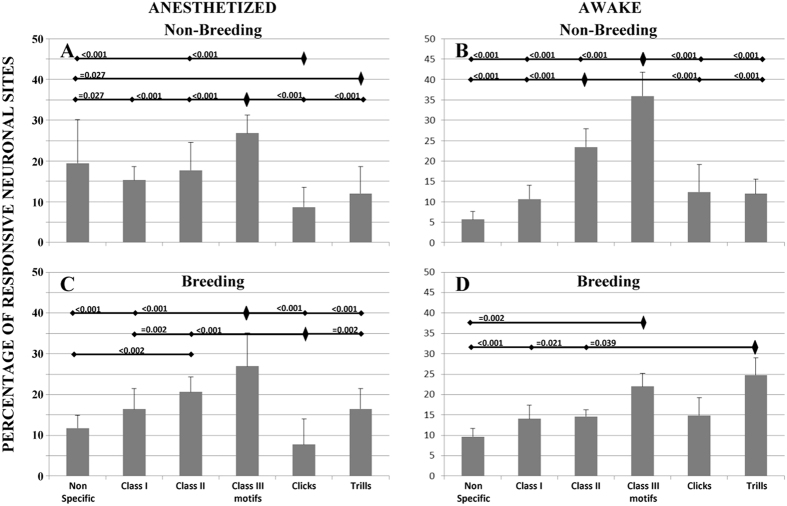
Neuronal preferences for song categories: percentage of responsive neuronal sites that responded to each class of acoustic stimulations in the anesthetized (**A**) or awake conditions (**B**) during the breeding season, and in the anesthetized (**C**) or awake conditions (**D**) during the non-breeding season. In the awake condition during the non-breeding season (B), there were clear differences between socially relevant stimuli (Class II and Class III motifs) and all the other types of stimuli that did not appear in the anesthetized condition. The p values are indicated above the type of stimulus when the differences are significant. Error bars indicate mean +− SD. The data shown on the right of the figure (**B,D**) are already published^15^.

**Figure 3:**
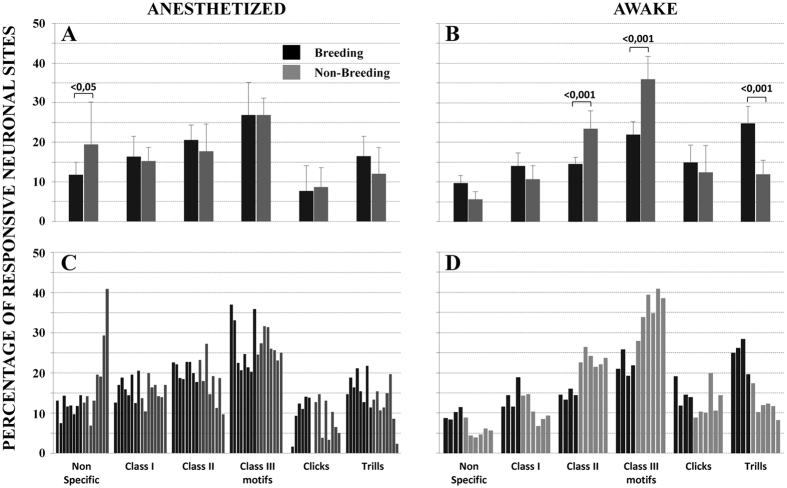
Seasonal variation in neuronal preferences: comparison of neuronal preferences between the breeding and non-breeding seasons in the anesthetized (**A**) and awake (**B**) birds: percentage of responsive neuronal sites i.e. of neuronal sites that responded to each class of acoustic stimulations. In the anesthetized condition, only an atypical change in response to non-specific stimuli appeared between breeding and non-breeding status while in the awake condition differences occurred for socially and sexually relevant stimuli. The percentage of responsive neuronal sites for each birds are shown in (**C,D**). The p values are indicated when the differences are significant. Error bars indicate mean +− SD. The data shown on the right of the figure (**B,D**) are already published^15^.

**Figure 4:**
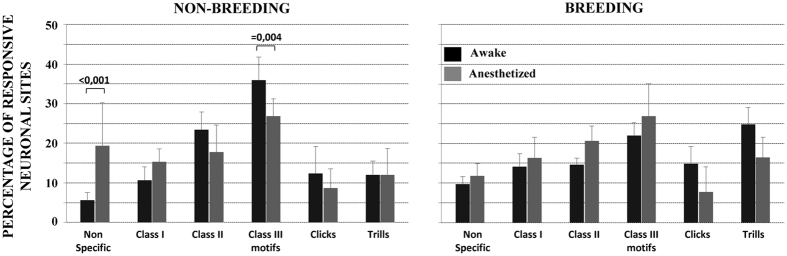
Effect of anesthesia at breeding (left) and non-breeding (right) season. There were statistical differences only at non-breeding season with an atypical change in responses to non-specific stimuli and a change in responses to socially relevant class III motifs. The p values are indicated when the differences are significant. Error bars indicate mean +/− SD.
